# Low Temperature Oxygen Activation on the NiAg(100) Single-Atom Alloy Surface

**DOI:** 10.1021/acs.jpcc.5c08033

**Published:** 2026-02-26

**Authors:** Cole A. Easton, Sarah M. Stratton, Nima Rajabi, Nishadi Amarathunga, Matthew M. Montemore, E. Charles H. Sykes

**Affiliations:** † Department of Chemistry, 1810 Tufts University, Medford, Massachusetts 02155, United States; ‡ Department of Chemical and Biomolecular Engineering, 5783 Tulane University, New Orleans Louisiana 70115, United States

## Abstract

Silver-catalyzed ethylene epoxidation remains the only industrially viable route for ethylene oxide (EO) production. However, this process requires chlorine and other promoters to achieve a high EO selectivity while still generating substantial CO_2_ emissions. A recent theory-guided approach identified Ni, in single-atom alloy (SAA) form, as a new promoter of this reaction. Specifically, the addition of Ni to Ag nanoparticles supported on α-Al_2_O_3_ at a highly diluted ratio (1 Ni per 200 Ag atoms) increased catalyst selectivity to EO by ∼25%, the same increase afforded by the ubiquitous industrial promoter chlorine. To better understand the effect of Ni, we investigated the interaction of O_2_ with NiAg(100) SAA surfaces by using scanning tunneling microscopy (STM) and density functional theory (DFT). While only molecular O_2_ was present when pure Ag(100) was exposed to O_2_ at 78 K, a distinct NiO_2_ species, indicative of O_2_ dissociation at Ni atom sites, was identified on the NiAg(100) SAA under the same conditions. High-resolution STM imaging backed by DFT simulations elucidated the formation of an O–Ni–O species with the oxygen atoms in 4-fold hollow sites. These findings provide direct experimental evidence that Ni atoms are very effective at O_2_ activation, even at cryogenic temperatures. This suggests that, in addition to the known role of Ni in stabilizing the unselective nucleophilic oxygen on Ag, it could also accelerate O_2_ dissociation, which can be rate limiting.

## Graphical abstract

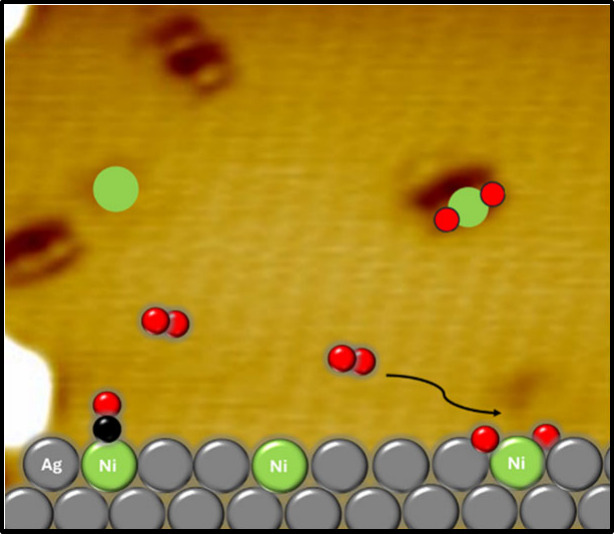

## Introduction

Ethylene epoxidation is an important industrial reaction (global market size of ∼40 billion USD annually) due to the high demand of its product, ethylene oxide (EO), which is a key intermediate in the production of a number of commodity chemicals.^1–4^ Ag-based particles supported on alumina facilitate all industrial EO production.^5–7^ However, Ag/*α-*Al_2_O_3_ catalysts have an inherent selectivity of only ∼50% toward EO with the remaining reacted ethylene producing CO_2_ and water.^8–10^ These catalysts rely on a series of promoters, primarily chlorine and elements like Cs and Re, to increase EO selectivity up to ∼90%.^3,10–14^ The use of chlorine can lead to corrosion, safety and environmental concerns, which has motivated research into alternative promoters.^15–18^ Given that CO_2_ is a byproduct of EO production, and the enormous scale at which this reaction is run, even minor improvements in EO selectivity could lead to significant reduction in CO_2_ emissions and energy costs.^5,15,19^ Both of these issues motivate continued efforts to understand and improve ethylene epoxidation on Ag-based catalysts.^1,2,8,20–30^

Recently, a joint theoretical, surface science, and catalytic study reported that the addition of trace amounts of Ni enhances the EO selectivity of Ag/*α-*Al_2_O_3_ catalysts by ∼25%, the same increase as Cl provides but without the need for constant coflow as is the case for Cl.^18^ Similar to the single-atom alloy (SAA) approach that led to new bimetallic combinations for (de)­hydrogenation reactions,^31–35^ density functional theory (DFT) calculations were used to screen a range of single atoms in Ag in search of a dopant that breaks scaling relationships, with both a low barrier for O_2_ activation and a relatively weak atomic oxygen binding energy.^18^ Ni was found to meet these criteria, and the surface structure and O_2_ activation were probed experimentally using surface science that then informed the design of Al_2_O_3_-supported NiAg nanoparticles that exhibited ∼80% selectivity toward EO under industrially relevant conditions without the presence of Cl.^18^ This selectivity increase was attributed to the stabilizing effect that Ni has on the unselective (nucleophilic) form of oxygen on Ag.

In terms of the active form of oxygen, some recent studies used a combination of spectroscopy and DFT modeling to investigate the Ag surface under ethylene oxidation conditions.^20,21^ A variety of oxygen species were detected in the active system, and dioxygen species on oxidized Ag were suggested to be the active and selective species for ethylene epoxidation. Given this, Ni may aid in oxidizing the surface and, thereby, stabilize these structures. Another recent study also found that an oxygen atom, as part of a dioxygen species on the Ag surface, is responsible for higher activity than lattice atomic oxygen and subsurface oxygen.^30^ However, this dioxygen species was not found to be exclusively selective toward EO formation, suggesting that the complex speciation of oxygen on Ag has other effects on selectivity, and there are more factors at play. In addition, this study demonstrated that, even in pure oxygen at 400 °C, the Ag surface remained majority metallic, in contrast to other referenced studies where oxidized Ag was considered ubiquitous under epoxidation conditions.

Due to the low sticking probability of oxygen on Ag(111) (10^–6^),^11,36^ surface science studies in ultrahigh vacuum (UHV) conditions using molecular oxygen are challenging.^37,38^ The more open Ag(100) facet has a higher sticking probability for O_2_ (∼10^–4^) as compared to the more commonly studied Ag(111),^11,36^ which provides the opportunity to study oxygen uptake without the need for high-pressure cells or reactive oxygen substitutes like O_3_ or NO_2_.^39,40^ Ag(100) has also been suggested to have inherent selectivity toward EO greater than other facets, which makes it a relevant system to study selective oxidation.^41–44^ Furthermore, the study of SAA active sites with scanning tunneling microscopy (STM) is generally easier on more open facets as the dopant atoms tend to alloy more uniformly on terraces as opposed to denser brims in the regions above step-edges seen on (111) surface facets.^45^

We used a combination of STM and DFT to study the effects of oxygen on NiAg(100) and Ag(100) at low temperature. We demonstrated facile activation of O_2_ at 78 K and resolved the atomic-scale structure of the resulting O–Ni–O sites, while comparing these results to bare Ag(100) which binds O_2_ molecularly under these conditions^46,47^ and only dissociates O_2_ above 150 K.^39^ Together with DFT calculations and STM image simulations, this study elucidates a potentially important O_2_ activation site on this promising new ethylene epoxidation SAA.

## Methods

### Scanning Tunneling Microscopy

78 K STM experiments were conducted in an ultrahigh vacuum (UHV) chamber with a base pressure of 1 × 10^–11^ mbar using a low temperature (LT)-STM (Omicron Nanotechnology). An Ag(100) single crystal (Princeton Scientific 99.999% purity <0.1 degree polish) was used for experiments. Sample cleaning was performed in a connected preparation chamber with a base pressure of <5 × 10^–10^ mbar. Repeated cycles of Ar (Airgas 99.99%) ion sputtering (1 keV, 10–20 μA) and annealing to 825 K were used to clean the Ag(100) crystals. STM images were obtained at 78 K after cryogenically cooling the STM stage. Oxygen (99.9% Middlesex gases) was dosed through high-precision leak valves. Ni was deposited via hot filament evaporation from a high-purity Ni rod at a sample temperature of 300 K. Coverages of the different species present were calculated as an average from ∼20 STM images comprising an area of ∼250 nm^2^ for each coverage through manual counting of features using an STM image processor (SPIP). Coverages are based on the number of specific features per image divided by the total number of Ag surface atoms in each image. Error bars on coverage were one standard deviation.

### Density Functional Theory

All DFT calculations were performed with the VASP code.^48,49^ A 400 eV cutoff was used for the plane-wave basis set. The PBE exchange–correlation functional was used,^50^ with the Tkatchenko–Scheffler method for dispersion corrections.^51^ A 7 × 7 × 1 k-point mesh was used for the 3 × 3 surface cells. Four layers were used with the bottom two fixed at their bulk positions. An electron convergence tolerance of 10^–5^ eV was used, along with a force tolerance of 0.03 eV/Å. The dimer method was used to locate transition states.^52^

## Results and Discussion

In order to investigate oxygen activation on the NiAg(100) SAA surface, we used 78 K STM and imaged the surface before and after the introduction of O_2_. First, a NiAg(100) SAA was synthesized by depositing ∼1% Ni on a clean Ag(100) surface at 300 K and then characterized with STM (Figure 1A–C). On the as-deposited NiAg(100) surface, three features are observable in STM: ejected silver islands (red arrows), CO-covered Ni atoms (orange arrows), and bare Ni atoms (green arrows). The ejected silver islands are formed when Ni atoms place exchange with surface Ag atoms at room temperature.^45^ The Ni atoms all occupy surface sites, directly replacing the surface Ag atoms. The CO-capped Ni atoms were identified using STM tip voltage pulse experiments that desorb the CO molecules from the top of the Ni sites. After these pulses, the appearance of the features was identical to that of bare Ni atoms in Ag(100).^53^ Examples of these single molecule CO desorption experiments are shown in Figure S1.

**Figure 1:**
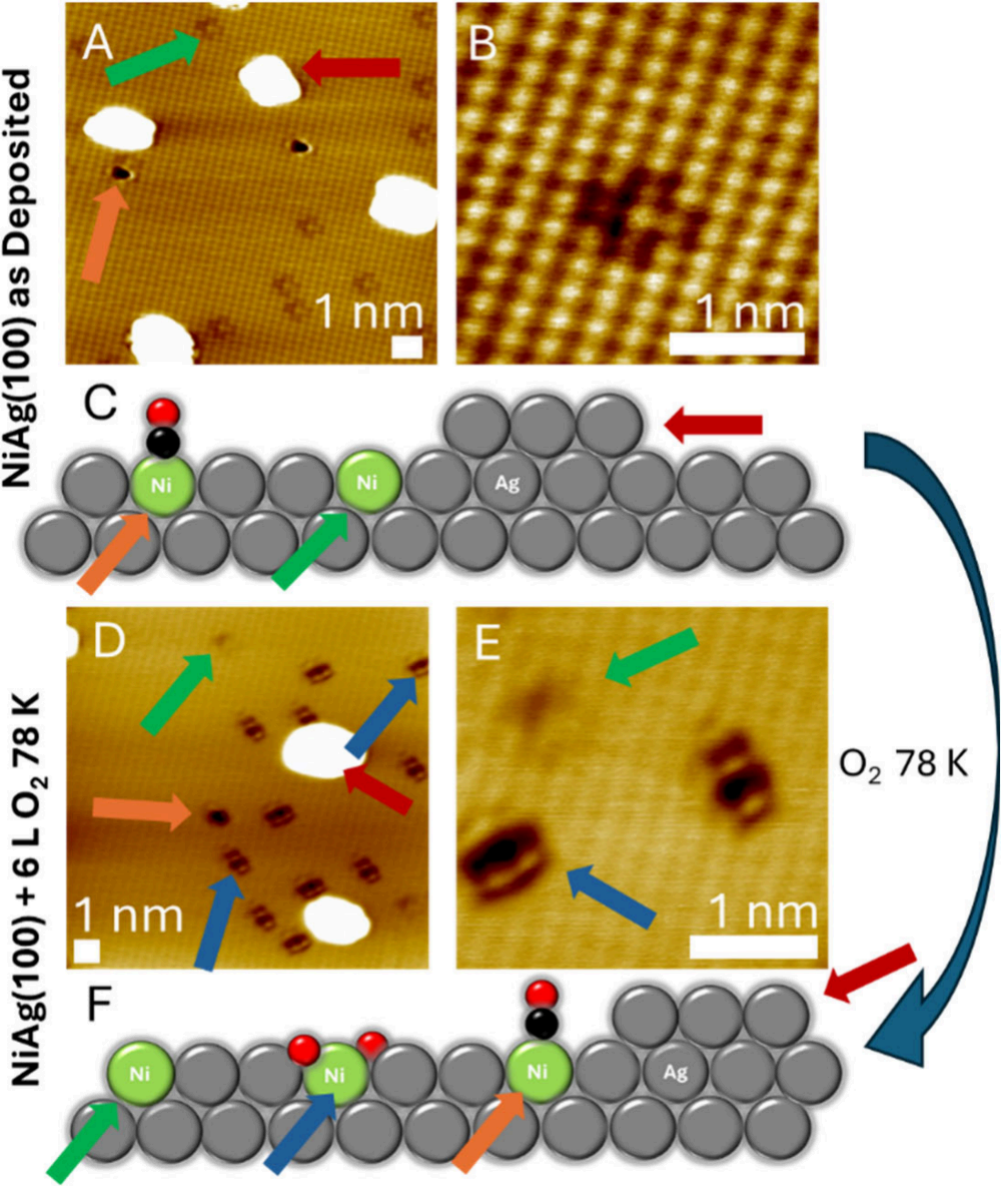
Overview of low temperature oxygen activation on 1% NiAg(100). A) Atomically resolved STM image of the as-prepared NiAg(100). Bare Ni single-atom sites are visible as “cross” features (green arrow), CO-capped Ni sites (orange arrow) appear as depressions, and ejected Ag islands are visible as white protrusions (red arrow). B) High-resolution STM image of a bare Ni single-atom site in Ag(100). The Ni atom occupies a site in the Ag lattice, replacing the Ag atom. C) Schematic of the NiAg(100) surface features with arrows that correspond to the STM images. D) STM image of NiAg(100) after exposure to 6 Langmuir (L) (1 L = 1 × 10^–6^ Torr·s) of O_2_ at 78 K. Bare Ni sites, CO-capped Ni sites and Ag islands are still visible. New features appear (blue arrow) as rectangular depressions corresponding to the O–Ni–O sites. E) STM image of bare Ni and O–Ni–O sites with atomic resolution. Imaging conditions were 10 mV and 1 nA for each image. All images were acquired at 78 K. F) Corresponding schematic of STM data in panels D and E.

To study the effects of oxygen dissociation, the samples were exposed to 6 L (1 L = 1 × 10^–6^ Torr/s) O_2_ at a surface temperature of 78 K. After O_2_ exposure, in addition to the features found on the as-deposited NiAg(100) surface, we observed a new species that appeared as rectangular depressions. This proposed nickel–oxygen species has two equivalent orientations, as would be expected on a square packed (100) surface as seen in Figure 1E. The mirror symmetry of the species is consistent with two O atoms bound to one Ni atom. Most significantly, the surface remained at 78 K during O_2_ exposure and subsequent imaging thereby demonstrating facile O_2_ dissociation.

To further interpret these experimental findings, DFT was used to calculate the energy barriers for O_2_ dissociation on NiAg(100) and Ag(100) (Figure 2). On bare Ag(100), the O_2_ activation barrier is 1.09 eV and the binding energy of the oxygen atoms is −0.87 eV. However, on the NiAg(100) SAA, the O_2_ dissociation barrier is only 0.26 eV with respect to adsorbed O_2_ and the binding energy of two O atoms bound at the Ni atom is stronger (−1.48 eV). Our DFT calculations revealed that isolated Ni atoms can stably bind both O atoms, whereas Ag prefers to bind only a single O atom per Ag atom (Figure S2).^46,54^ The barrier height is much higher on the pure Ag surface, which explains why oxygen only adsorbs molecularly at 78 K on Ag(100) as we describe later in the paper.^55,56^ The low O_2_ dissociation barrier on the NiAg(100) SAA, demonstrated with experiment and theory, is significant as O_2_ dissociation is the rate-limiting step in many selective oxidation reactions on Ag-based heterogeneous catalysts.^5,9,18,57^ Ni also binds oxygen 0.6 eV more strongly than Ag, which explains the lack of O atoms bound to Ag sites at 78 K.

**Figure 2:**
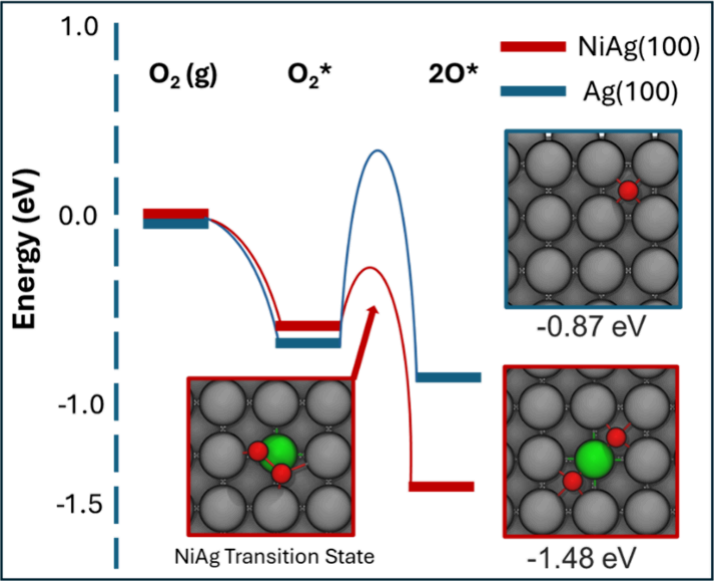
Oxygen activation energetics calculated by DFT. The activation barriers, transition states, and stabilities/reaction energies are shown for both NiAg(100) SAA (red, defined as isolated dopant Ni sites in the Ag surface) and Ag(100) (blue). The activation barrier and transition state energy for O_2_ on bare Ag(100) are much higher than on NiAg(100). Additionally, Ni prefers to bind two oxygen atoms rather than a single oxygen atom as is the case for bare Ag.

In order to quantify these effects and compare them to Ag(100), further STM experiments were performed and the results are shown in Figure 3. First, the aforementioned control experiment during which 6 L of O_2_ were dosed onto a clean Ag(100) surface while holding it at 78 K was performed (Figure 3A,B). As expected from the high barrier for O_2_ dissociation computed for Ag(100), no features were observed after exposure of the surface to O_2_. While molecular O_2_ can physisorb on Ag(100) at 78 K, its fast diffusion at this temperature led to featureless STM images as shown in Figure 3B.^47,55,56^ In contrast, NiO_2_ features appeared in STM images after the same oxygen exposure on NiAg(100), confirming the much lower O_2_ dissociation barrier on the SAA (Figure 3C,D).

**Figure 3:**
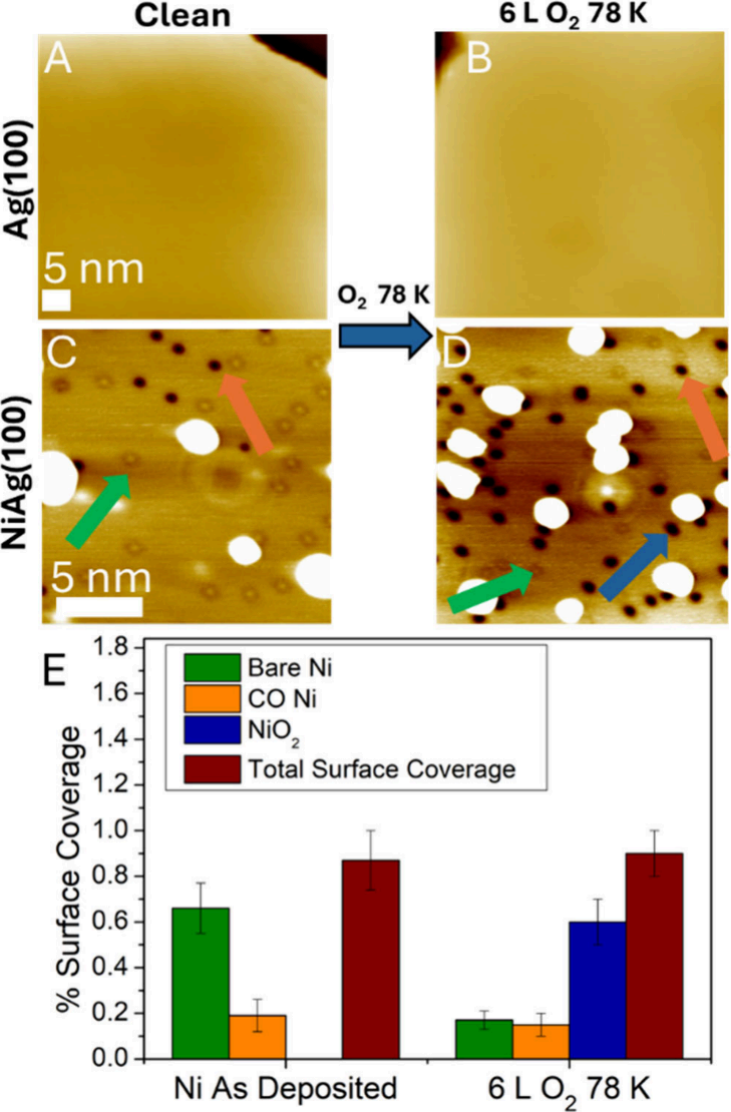
STM comparison of oxygen deposition on (A,B) Ag(100) held at 78 K and (C,D) NiAg(100) held at 78 K. A) STM image of the clean Ag(100) surface. B) STM image of the Ag(100) surface after exposure to 6 L O_2_ at 78 K. Molecular O_2_ cannot be imaged due to fast diffusion.^47,55,56^ C) STM image of the NiAg(100) surface as deposited. Three features are observable: Bare Ni atoms (green arrow), CO-capped Ni atoms (orange arrow), and islands of ejected Ag formed during the alloying of Ni with Ag (bright white features). These displaced Ag islands appear larger than expected given the small amount of Ni deposited because the STM tip is not infinitely sharp, which leads to topographically higher features appearing wider than they are. D) STM image of NiAg(100) after exposure to 6 L O_2_ at 78 K. Typical scanning conditions were 100 mV and 300 pA. STM imaging was performed at 78 K. E) Quantification of the different species observed in STM on NiAg(100) before and after 78 K oxygen exposure. Error bars are single standard deviation. The NiO_2_ species could be easily distinguished from the CO-capped Ni atoms using high bias voltage scanning conditions to desorb the CO molecules but not the NiO_2_ (Figure S3).

The distribution of species present on the NiAg(100) surface before and after the exposure to O_2_ was quantified with STM (Figure 3E). On the as-deposited surface, 0.8% ML Ni was present as either bare Ni atoms (0.6%) or CO-capped Ni atoms (0.2%). Upon introduction of oxygen, the amount of CO-capped Ni atoms remained constant, while the number of bare Ni sites decreased and the new NiO_2_ species increased by the same amount. These results further support the idea that these features arise due to dissociative adsorption of oxygen at the Ni atom sites and that O_2_ cannot displace the CO bound to Ni atoms at this temperature.

To further support the identity of the proposed structures, DFT-simulated STM was combined with atomic-resolution experimental STM images (Figure 4). Figure 4A,B shows the O-NiAg(100) system under atomic resolution (A) and typical (B) STM imaging conditions. With typical resolution, the bare Ni sites are less defined (green arrows) but still observable, and the NiO_2_ sites appear somewhat rectangular in shape with two orientations (blue arrows). The two diagonal NiO_2_ species have slightly different appearances in the atomic resolution images due to convolution with the STM tip shape (see also Figure S8).^58^ Figure 4C shows a DFT-simulated STM image of the NiO_2_ species. Oxygen–nickel species with different stoichiometries and structures are shown in Figure S4. This simulated NiO_2_ (where the bright areas are oxygen atoms, and the Ni atom is gray in the center) is similar to the oxygen feature observed with STM. In particular, the orientation of the species and the location of its bright and dark areas are in good agreement and support our assignment as NiO_2_.

**Figure 4:**
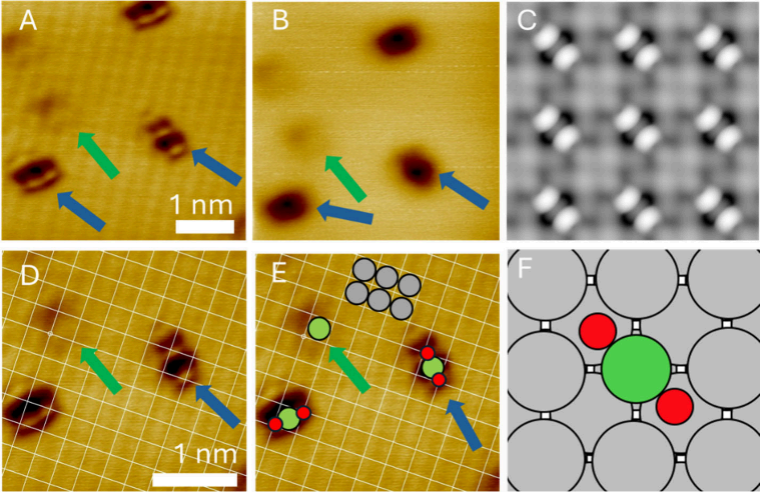
DFT-simulated STM and experimental atomic-resolution images. A) 78 K STM image of the NiAg(100) surface after exposure to O_2_. Bare Ni sites appear as cross-shaped features (green arrow) and NiO_2_ sites as rectangular features that run diagonally to the (100) lattice (blue arrows). Imaging conditions were 10 mV and 1.0 nA. B) The same area was imaged under typical STM resolution (300 mV, 1 nA). Bare Ni sites appear to be fainter than in A but retain the same shape. NiO_2_ sites appear as depressions under these conditions but retain their rectangular shape. C) Simulated STM image of O–Ni–O features on Ag(100). The bright areas are oxygen atoms, while the central gray area is a Ni atom. D, E) Atomic resolution 78 K STM image of the system with a grid showing the 4-fold hollow sites of the Ag(100) lattice. Panel E shows the surface with Ni and O atoms overlaid as calculated with DFT. The locations of Ni (green), oxygen (red), and silver (gray, only some Ag atoms are highlighted) sites are shown. Imaging conditions were 10 mV and 1 nA. F) DFT-calculated relaxed geometry of dissociated O_2_ on NiAg(100) SAA. The oxygen atoms sit in 4-fold hollow sites each side of the Ni dopant atom.

To test if the O–Ni–O assignment is consistent with the size and orientation of our observed species, we overlaid the Ag(100) lattice on an atomic resolution STM image (Figure 4D,E). The DFT-calculated adsorption site for oxygen on NiAg (Figure 4F) was used as a reference. DFT calculations and the atomically resolved STM images demonstrate that O atoms bind in 4-fold hollows on both Ag(100) and the NiAg(100) SAA, in agreement with the literature for Ag(100).^46,54,57^ Figure 4E shows both orientations of the NiO_2_ species. The brighter areas of the feature align with 4-fold hollow sites on opposite sides of a surface Ni atom. This structure agrees with our DFT geometry and simulated STM and provides further evidence for the formation of a NiO_2_ species with an O–Ni–O structure at 78 K on NiAg(100).

## Conclusions

These results demonstrate that isolated Ni atoms substitutionally alloyed on the surface of Ag(100) are highly active for low-temperature O_2_ activation and stabilize a well-defined O–Ni–O species. Using 78 K STM and DFT, we directly visualized dissociated oxygen bound in 4-fold hollow sites flanking individual Ni atoms and showed that these NiO_2_ features form under conditions where Ag(100) binds only molecular O_2_. Quantitative analysis established that O_2_ dissociation occurs selectively at bare Ni sites without displacing CO from Ni, while DFT revealed that Ni both dramatically lowers the O_2_ dissociation barrier relative to Ag and binds atomic oxygen more strongly than Ag, thereby preventing spillover at cryogenic temperatures. By resolving the local structure and energetics of these O–Ni–O motifs, this work provides molecular-level evidence that Ni-atom dopants can create highly active oxygen activation sites on Ag surfaces. This observation is significant given that O_2_ dissociation can be rate limiting in the ethylene epoxidation reaction, and many reactor studies have demonstrated that under ethylene epoxidation conditions the rate order is positive in oxygen and small or negative in ethylene.^59^ This points toward the O_2_ activation step being important in the rate of ethylene epoxidation. Furthermore, ethylene epoxidation catalysts are run at relatively low temperatures in order to minimize secondary combustion of the as-formed ethylene epoxide to CO_2_. These lower temperatures lead to conversions of ∼10%, and increasing temperature results in a decrease in selectivity. Therefore, if the oxygen dissociation step can be catalyzed by Ni atoms in Ag, then catalysts may be run at lower temperature with Ni aiding in the supply of oxygen and thereby achieve higher conversions without compromising selectivity. This could work in concert with the known effect of Ni enhancing selectivity by stabilizing unselective (nucleophilic) oxygen.^18^

## Supplementary Material



## Notes

STM images of Ni alloying, oxidation and high-resolution images, DFT calculations of oxygen binding energies and simulated STM images (PDF)
