# Supplementary material for: Low Temperature Oxygen Activation on the NiAg(100) Single-Atom Alloy Surface
Source: J Phys Chem C Nanomater Interfaces. 2026 Feb 26;130(10):3789–94. doi: 10.1021/acs.jpcc.5c08033 (PMC12990106; doi:10.1021/acs.jpcc.5c08033)
Supplement: Supplementary file 1 [file jp5c08033_si_001.pdf]

Supporting Information

Low Temperature Oxygen Activation on the NiAg(100) Single-Atom Alloy Surface

Cole A. Easton,[1] Sarah M. Stratton,[2] Nima Rajabi,[1] Nishadi Amarathunga,[1] Matthew M. Montemore,[2] and E. Charles H. Sykes.[1*]

[1]Department of Chemistry, Tufts University, Medford, Massachusetts 02155, United States

[2]Department of Chemical and Biomolecular Engineering, Tulane University, New Orleans Louisiana 70115, United States

*charles.sykes@tufts.edu *mmontemore@tulane.edu

Supplementary Figures

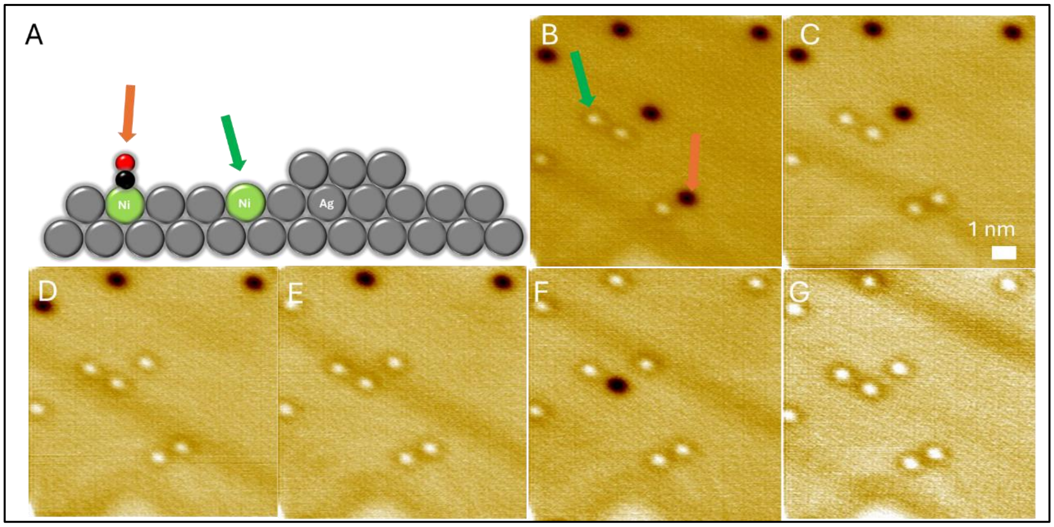

Figure S1. STM Pulse Sequence of CO molecules on NiAg(100). A) Depiction of NiAg(100) surface after Ni deposition. CO-capped Ni (orange arrows) and bare Ni atoms (green arrows) are present). B-G) 78 K STM of a 5.0 V pulse sequence where CO molecules are desorbed one-by-one from Ni atoms in the surface of NiAg(100). CO-bound atoms appear as depressions and bare Ni atoms appear as donut-shaped protrusions. In F, a CO molecule readsorbed to a previously bare Ni atom. Scanning conditions were -100 mV and 800 pA, and a scale bar is shown in C.

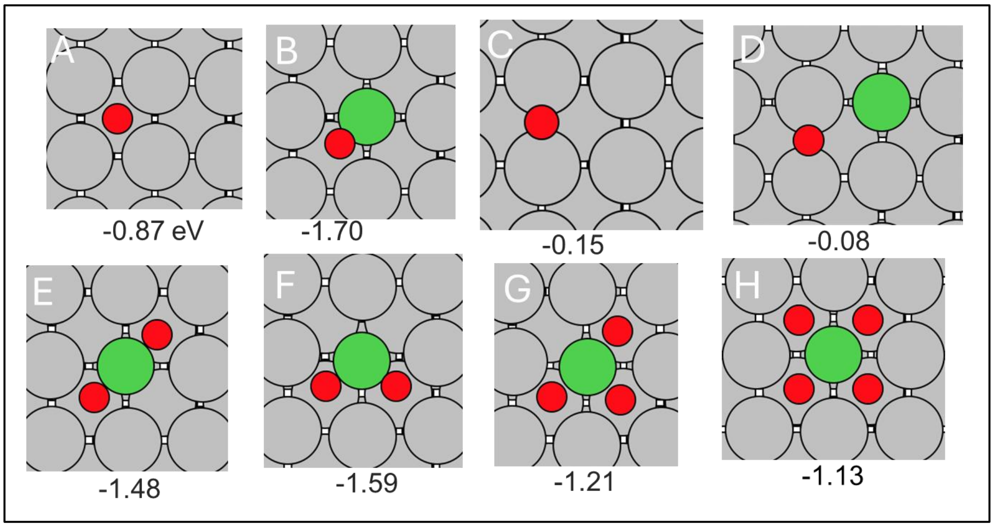

Figure S2. DFT-calculated binding sites and associated adsorption energies for oxygen atoms in various configurations on Ag(100) and NiAg(100). For 2O/NiAg, the structure observed experimentally is apparently not the most favorable configuration predicted by DFT calculations. We attribute this primarily to the fact that DFT can be inaccurate for transition metal oxides. As the surface becomes more oxide-like, we expect larger errors. Other factors, such as entropy effects, are likely to be smaller but could play a role.

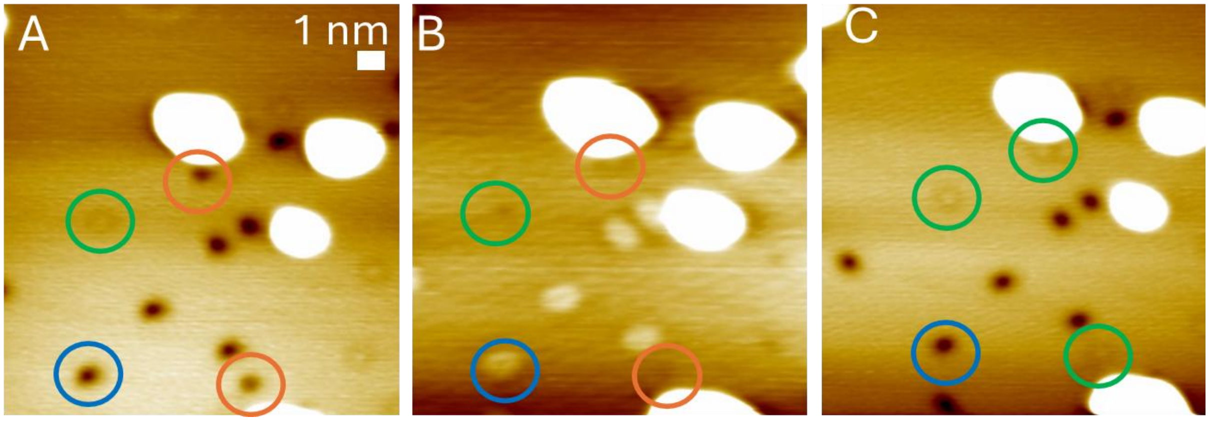

Figure S3. Higher bias voltage scanning on O-NiAg(100). A) STM of NiAg(100) after O_2 exposure at 78 K. Bare Ni atoms (green circle donut protrusion), O-Ni-O sites (blue circle, rectangular depressions) and CO-bound Ni atoms (orange circles, small depressions) are all present. Scanning conditions were 100 mV 300 pA. B) 3.0 V (300 pA) scan of the same area. Ni atoms now appear as faint depressions. O-Ni-O appears as rectangular shallow protrusions. The CO bound to Ni atoms have been desorbed due to the high voltage. C) 100 mV scan (300 pA) after two 3 V scans. The appearances of the various species are the same as in A. However, now there are no CO-bound Ni atoms. Because the only depressions present are O-Ni-O sites, and the Ni atoms previously under CO are visible as additional protrusions, we can distinguish between the two species and count the STM features to determine surface coverage. By counting the total number of depressions and protrusions before and after high-voltage scanning, we are able to deduce surface coverages for each species.

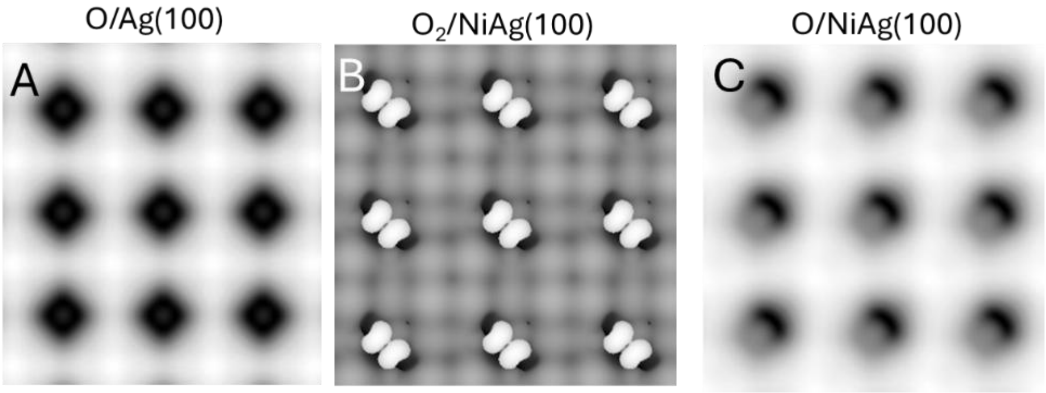

Figure S4. Simulated STM of other species. A) Simulated STM of O-Ag(100) in which O adatoms appear as square depressions in four-fold hollows. B) Simulated STM image of O_2 molecules on NiAg(100). The oxygen molecules appear as protrusions with slight depressions on their edges. C) Simulated STM image of single O adatoms on Ni atoms in NiAg(100). Ni appears gray and the oxygen adatom is bound in the four-fold hollow and appears as a small depression. Each of these species has a different appearance than the O-Ni-O species observed in this work, proving further support of the assignments in the main paper.

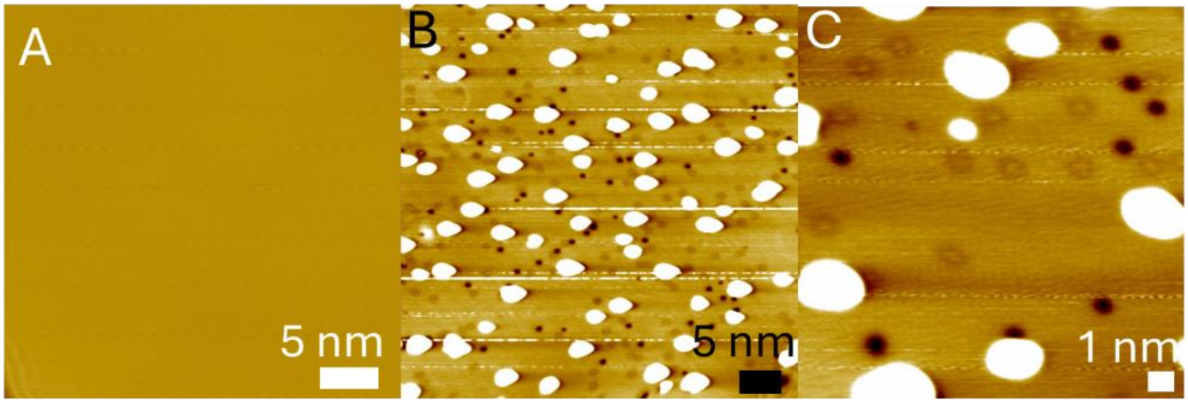

Figure S5. STM of the Ag(100) surface before and after Ni deposition. A) Bare Ag(100) surface after cleaning. No impurities or other species are present. B) Ag(100) after deposition of ~1% ML Ni on Ag(100). Ejected Ag islands, CO-bound Ni and bare Ni atoms are present. C) Zoom of the NiAg(100) surface. Imaging conditions were 300 mV and 300 pA at 78 K.

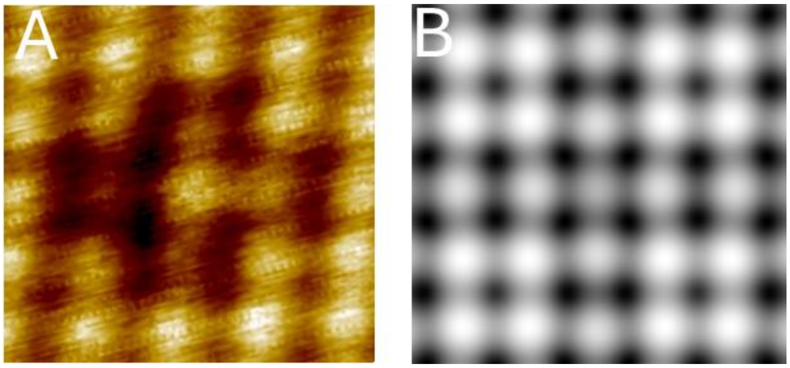

Figure S6. Experimental and simulated STM images of isolated Ni atom in NiAg(100). A) Atomic-resolution STM of an isolated Ni atom in the NiAg(100) surface. Scanning conditions were 10 mV and 1 nA, and the image is 1.5 by 1.5 nm^2. B) DFT-based simulated STM image of a Ni site (gray atom in the center) in NiAg(100). Both images share the same "cross" shape with four adjacent Ag atoms perturbed by the Ni atom in the center.

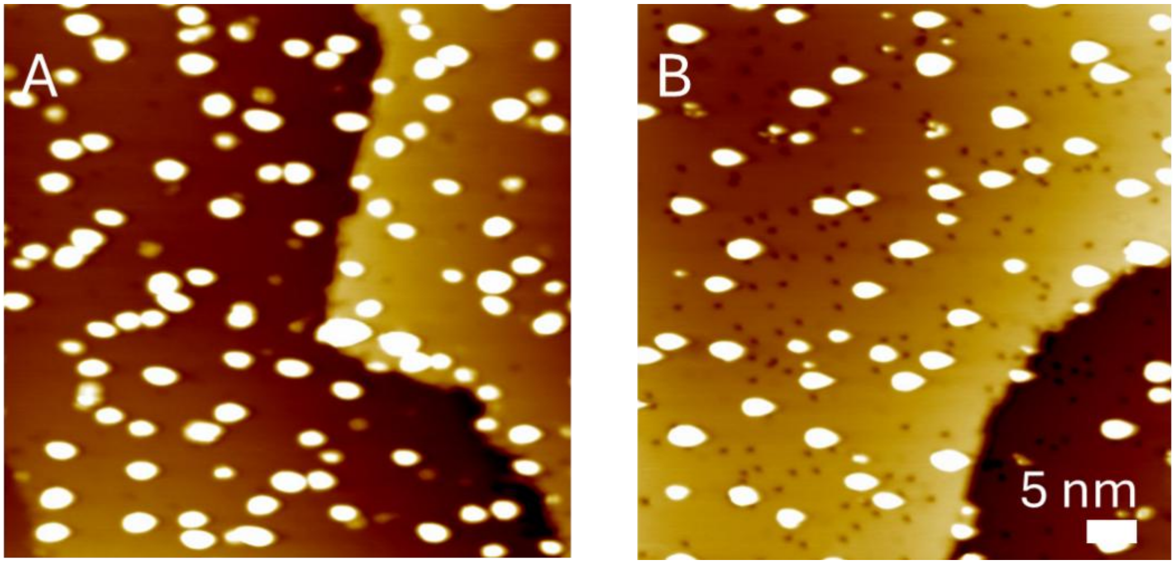

Figure S7. STM images comparing step edges on NiAg(100) and O-NiAg(100). A) Step edge on NiAg(100) as deposited. Bare Ni sites, Ag islands and CO-bound Ni sites are present. There is no clustering of Ni at the step edge. B) Step edge on NiAg(100) after exposure to 6 L O_2 at 78 K. Bare Ni sites, Ag islands, CO-bound Ni sites and O-Ni-O species are present. No clustering of Ni or O was observed at the step edges. Scanning conditions were 100 mV and 300 mA for each image. Images are 50 by 50 nm^2, and a scale bar shown in B.

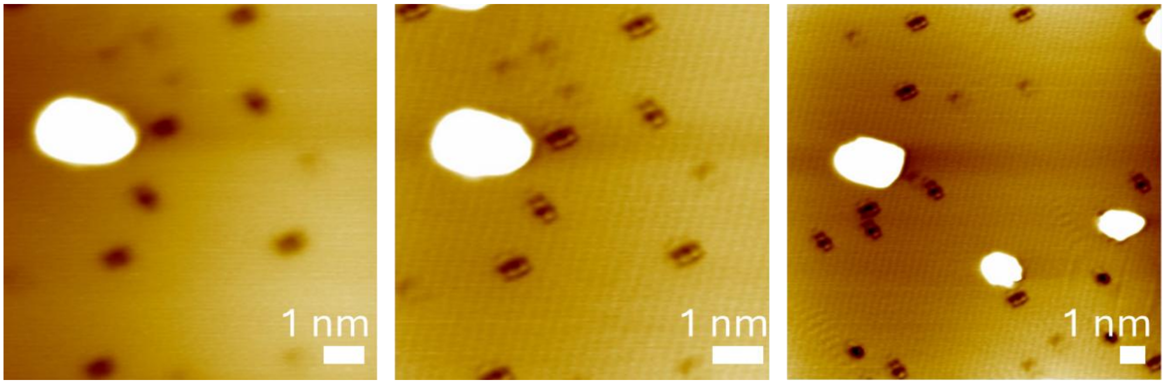

Figure S8. Additional 78 K STM Images. A) STM image of NiAg(100) after 6 L O_2 exposure under typical resolution. Scanning conditions were 100 mV and 1 nA. B) Same area under atomic resolution. Scanning conditions were 10 mV and 1 nA. C) Separate area under atomic resolution. Scanning conditions were 10 mV and 1 nA.

References

(1) Kresse, G.; Furthmüller, J. Efficiency of Ab-Initio Total Energy Calculations for Metals and Semiconductors Using a Plane-Wave Basis Set. *Comp. Mat. Sci.* **1996**, *6* (1), 15–50. https://doi.org/10.1016/0927-0256(96)00008-0.

(2) Kresse, G.; Hafner, J. *Ab Initio* Molecular Dynamics for Liquid Metals. *Phys. Rev. B* **1993**, *47* (1), 558–561. https://doi.org/10.1103/PhysRevB.47.558.

(3) Perdew, J. P.; Burke, K.; Ernzerhof, M. Generalized Gradient Approximation Made Simple. *Phys. Rev. Lett.* **1996**, *77* (18), 3865–3868. https://doi.org/10.1103/PhysRevLett.77.3865.

(4) Tkatchenko, A.; Scheffler, M. Accurate Molecular Van Der Waals Interactions from Ground-State Electron Density and Free-Atom Reference Data. *Phys. Rev. Lett.* **2009**, *102* (7), 073005. https://doi.org/10.1103/PhysRevLett.102.073005.

(5) Heyden, A.; Bell, A. T.; Keil, F. J. Efficient Methods for Finding Transition States in Chemical Reactions: Comparison of Improved Dimer Method and Partitioned Rational Function Optimization Method. *J. Chem. Phys.* **2005**, *123* (22), 224101. https://doi.org/10.1063/1.2104507.
